# How psychological ownership over nutritional products affects purchase intentions of high-pressure working groups

**DOI:** 10.3389/fnut.2024.1401035

**Published:** 2024-08-08

**Authors:** Boyu Liang, Dajun Yang, Fuqiang Tan, Dajun Sun, Jianshu Li

**Affiliations:** ^1^College of Art, Hebei University of Economics and Business, Shijiazhuang, Heibei, China; ^2^School of Administration, North Sichuan Medical College, Nanchong, Sichuan, China; ^3^Research Center for Industry Digitalization, Huainan Normal University, Huainan, Anhui, China

**Keywords:** psychological ownership over nutritional products, high-pressure working groups, purchase intentions, nutritional awareness, nutritious foods

## Abstract

This study aims to investigate the influence of psychological ownership of nutritional products on the purchase intention of high-pressure working groups, as well as the underlying mechanisms and boundary conditions of this influence. This study aims to investigate the impact of psychological ownership of nutritional products on the purchase intention of high-pressure working groups, as well as the underlying mechanisms and boundary conditions of this influence. The research contributes through the use of variance analysis, mediation models, and moderation models on data from adult participants over the age of 18, across three experiments. Experiment 1, conducted on the Credamo platform, randomly recruited 285 participants, with 148 males (51.9%) and 137 females (48.1%), and the results indicated a direct impact of psychological ownership on purchase intention. Building upon this, Experiment 2, which also utilized the Credamo platform and recruited 280 participants consisting of 136 males (48.6%) and 144 females (51.4%), further revealed the mediating role of perceived value. Experiment 3, with 265 participants randomly recruited on the Credamo platform, including 131 males (49.4%) and 134 females (50.6%), identified the moderating effect of nutritional awareness. The theoretical contribution of this study lies primarily in its in-depth exploration of the impact of psychological ownership of nutritional products. By integrating the factors of perceived value and nutritional awareness, it provides a systematic explanation for better understanding the purchase intentions of high-pressure working groups. Additionally, this study offers valuable strategies for merchants to enhance the purchase intentions of high-pressure living groups.

## 1 Introduction

A survey conducted by the China Youth Daily Social Survey Center in collaboration with the Questionnaire Network, which polled 2001 participants, revealed that 71.0% of respondents believe that young people who are currently striving in the workforce face numerous issues with nutritional deficiency. The respondents identified fast-paced lifestyles (69.4%), high levels of psychological stress (46.9%), and a lack of time for proper meals (45.8%) as the primary causes. Evidently, within the context of a fast-paced and highly competitive society, individuals in high-pressure work environments frequently encounter a variety of challenges and difficulties (1). The high demands of the job, combined with long working hours and constant stress, can damage their physical and mental health (2, 3). As a result, in these high-pressure work environments, many people are increasingly turning to nutritional products and supplements to fulfill their dietary needs and maintain optimal health (4, 5). While the importance of nutritional products in maintaining overall health has been widely recognized, a large part of previous research has neglected the specific nutritional needs of individuals working under high pressures. Most research in the field of nutrition and health has focused on the general population or specific groups, such as athletes or the elderly, while ignoring the unique dietary needs of people with high-pressure work (6). Understanding the psychological ownership over nutritional products and its impact on the purchase intentions of high-pressure working groups is crucial, which could help to develop effective marketing strategies to cater to their specific needs. By investigating the factors that affect their decision-making process, this study aims to explore the impact of psychological ownership over nutritional products on the purchase intentions of high-pressure working groups and its internal mechanisms, as well as providing new ideas for marketing the nutritional products and addressing the needs of high-pressure working groups for nutritional product.

While previous research has shed light on the importance of addressing the nutritional needs of individuals working with high-pressure, there is still a noticeable gap in understanding how psychological ownership over nutritional products affects their purchase intentions (7). In recent years, there has been increasing academic researcher interest in exploring the needs of high-pressure working groups for nutritional products, where high levels of stress often lead to irregular diets, reliance on convenience foods, and inadequate nutrient intake (8). As a result, these individuals may be at higher risk for nutritional deficiencies and related health problems (9). Academic researchers have focused on the need for nutritional products in high-stress working groups, for example, a study of Kiecolt-Glaser (10) suggests that work-related stress triggers inflammation in the body, which makes the intake of nutritional products extremely important. However, there is limited research on the purchase intentions of high-stress working groups. Whether purchase intentions of high-pressure working groups are affected by psychological ownership over nutritional products has not received much attention from academic researchers, and its theoretical and marketing value has not been well recognized. This oversight can be damaging, as understanding the role of psychological ownership can provide valuable insights into the motivations and decision-making processes of this particular group (11). However, in terms of the current literature, the mechanisms for the psychological ownership over nutritional products affecting the purchase intentions of high-pressure working groups are not clear. Therefore, we need to answer the following question: does psychological ownership over nutritional products have a positive impact on the purchase intentions of high-pressure working groups? What are the underlying mechanisms by which psychological ownership of nutritional affects the purchase intentions of high-pressure working groups?

To address the gaps in the above research, this study developed a theoretical framework to discuss whether and how psychological ownership over nutritional products can have a positive impact on the purchase intentions of high-pressure working groups. Guided by psychological ownership theory, this study argued that psychological ownership over nutritional products can influence the purchase intentions of the high-pressure working groups. Psychological ownership of brands has been proven by several studies to influence individual psychological states (11). However, previous research has focused on the connection between psychological ownership of product brands on the consumption behavior of high-pressure working groups, and there has been a lack of attention on how psychological ownership over nutritional products affects their consumption behavior. Based on the above, we firstly proposed that there is an impact of psychological ownership over nutritional products on the purchase intention of high-pressure working groups; second, we explored the mediating role of perceived value on the relationship between psychological ownership over nutritional products and the purchase intention of high-pressure working groups from the perspective of the perceived value for nutritional products; last, based on the perspective of the nutritional awareness of high-pressure working groups, we proposed the moderating role of nutritional awareness, which means that nutritional awareness moderates the relationship between psychological ownership over nutritional products and purchase intention of high-pressure working groups.

This study makes the following important contributions to nutritional product marketing and consumer behavior in high-pressure working groups. Although research on psychological ownership over nutritional products is relatively common, few studies have focused on the function of psychological ownership over nutritional products and its impact on purchase intentions of high-pressure working groups. Therefore, this study is an earlier attempt to link psychological ownership over nutritional products and purchase intention of high-pressure working groups, which further explores the internal mechanisms and boundary conditions between the two and enriches the existing literature on nutritional products marketing and consumption behavior of high-pressure working groups (12). Second, this study verified the mediating role of perceived value. The expectation and satisfaction of high-pressure working group for the product can enhance their perceived ownership and control of the nutritional product, which can further increase their purchase intention. Thus, the internal mechanism between psychological ownership over nutritional products and purchase intention of the high-pressure working group can be better elaborated, which enriches the research related to the perceived value theory (13). Last, this study validated the moderating role of nutritional awareness. It indicated that the focus of high-pressure working group on the nutritional value and health factors of nutritional products, could enhance their purchase intentions. Thus, the theoretical contribution of this study is mainly to provide a systematic understanding of the psychological ownership over nutritional products, and how to combine it with the perceived value and nutritional awareness of high-pressure working groups, to affect their purchase intentions. In fact, this study also provided valuable measures for merchants to increase the purchase intention of high-pressure working groups.

## 2 Theoretical framework and hypotheses

### 2.1 Psychological ownership

Psychological ownership is the right of individuals to subjectively feel and control their thoughts, emotions and consciousness (14). It is individuals’ perception and experience of independence and autonomy over their inner world. Pierce et al. (15) developed the concept of psychological ownership. The development of psychological ownership can be divided into three stages. The first stage is the stage of perception, where individuals begin to realize that they have independent thoughts and emotions, and are capable for controlling and management (11). The second the stage of emotions, where individuals begin to realize that their feelings and emotions are independent of others and that they are able to express and manage them autonomously (16). The third stage is the stage of consciousness, in which individuals begin to recognize that their consciousness and conscious activities are independent of others and are able to choose and control them autonomously.

Psychological ownership has a significant impact on consumer behavior. First, psychological ownership influences individuals’ choice and consumption behavior for products and services (11). Individuals tend to choose those products and services that meet their needs and preferences as a way to acknowledge their psychological ownership. Second, psychological ownership influences individuals’ use and consumption behavior of products and services (17). Individuals tend to use and consume products and services in their own way to acknowledge their psychological ownership. Again, psychological ownership influences individuals’ evaluation and satisfaction with products and services (7). Individuals tend to show higher appreciation and satisfaction for products and services that acknowledge their psychological ownership. Last, psychological ownership promotes the needs for personalized products and services (18). Individuals tend to choose products and services that satisfy their unique needs, which enhances their satisfaction and loyalty to the products and services.

To sum up, by increasing individuals’ sense of identity, control and personalized needs, psychological ownership enhances their satisfaction and loyalty to products and services. It is obvious that psychological ownership is the right of individuals to subjectively feel and control their thoughts, emotions and consciousness, which has been applied in many fields and has significant impact on consumer behavior (19).

### 2.2 Psychological ownership over nutritional products and purchase intentions of high-pressure working groups

The high-pressure working group refers to people who are exposed to high levels of stress and pressure at work, and who are often required to take on a large number of tasks and responsibilities, with long working hours and often excessive overtime (1). In high-pressure work environments, people are often prone to physical and psychological fatigue and stress (20). In order to cope with these problems, some people choose to purchase nutritional products to improve their health and productivity.

This study argued that psychological ownership over nutritional products refers to individuals’ psychological feelings and perceptions about purchasing and using nutritional products, which includes individuals’ needs, expectations, beliefs, attitudes, and behaviors toward nutritional products (21). Psychological ownership over nutritional products has a significant impact on the purchase intention of high-pressure working groups. First, high-pressure work groups tend to be concerned about their health status (22). They are able to realize the importance of their physical condition for their productivity and quality of life. Therefore, they are more open to and willing to purchase nutritional products for improvement. Second, the high-pressure working groups tend to have high expectations of the efficacy and effectiveness of nutritional products (23). They want nutritional products to improve their physical strength and energy, reducing fatigue and stress. Therefore, they are more likely to purchase nutritional products that are advertised as being capable of providing these effects. In addition, the beliefs of high-pressure working groups about nutritional products may also affect their purchase intentions (24). Some people believe that nutritional products can help them stay healthy and slow down aging, thereby improving productivity and quality of life. This belief will motivate them to purchase and use those nutritional products.

However, high-pressure working groups also consider the following factors when purchasing nutritional products. First, they are usually more sensitive to the price of nutritional products. Due to their higher income, high-pressure working groups are more inclined to purchase higher-end nutritional products with higher prices, because they believe that they are of better quality (25). Second, the high-pressure working groups have higher demands on the safety and reliability of nutritional products. They are more likely to purchase brands and products that are certified to ensure their quality and safety (26). Last, the purchase intentions of high-pressure working groups are also affected by social factors. They usually pay attention to the consumption of their colleagues and friends, and will be more inclined to buy nutritional products that are used by people around them.

To sum up, the purchase intentions of the high-pressure working groups are affected by their psychological ownership over nutritional products. The purchase intention in nutritional products could be influenced by concerns about their health status, expectations about the efficacy and effectiveness, and beliefs, as well as their considerations about price, safety, and social factors. Therefore, manufacturers and distributors of nutritional products should learn about the needs and psychology of the high-pressure working groups and provide products that meet their expectations and requirements, in order to increase their purchase intentions (27).

Based on the above analyses, the following hypothesis was proposed.

*H1*: Psychological ownership over nutritional products enhances purchase intentions of high-pressure work groups.

### 2.3 The mediating role of perceived value

Perceived value refers to the actual or potential benefits and satisfaction that consumers perceive from products or services, and it is one of the most important factors that are considered by consumers, in the purchasing decision-making process, which can influence consumers’ purchase intentions and behaviors (28). Perceived value can affect individuals’ behavior and decision-making because people tend to prioritize those things that they perceive to have higher value (29). Perceived value can also be influenced by external factors, such as social culture and media publicity (30).

The impact of perceived value on consumers’ purchase intention is mainly reflected in the following aspects. First, the functional value of products or services (31). Consumers will evaluate whether products or services can satisfy their needs based on their functional characteristics. If the product or service can provide better functional value, consumers are more likely to purchase it. Second, the economic value of the products or services (7). Consumers will consider whether the price is reasonable and whether it provides economic benefits. If the economic value of products or services is high enough, consumers will be more motivated to purchase it. Third, the emotional value of the products or services (32). Consumers will assess the value of products or services based on the emotional experience they create. Consumers are more likely to purchase products or services when they bring pleasure, satisfaction, or enjoyment to them. Last, the social value of the products or services. Consumers will consider whether the products or services are consistent with social values, and whether they enhance their social status. Product or services with higher social value are more likely to be purchased.

Nutritional products are special products whose perceived value has a significant impact on the purchase intention of high-pressure working groups. High-pressure working groups usually face greater work pressure and physical health problems, so they have a higher demand for nutritional products (33). The perceived value of nutritional products affects the purchase intention of high-pressure working groups in the following aspects. From the perspective of the function in health care, the high-pressure working group has a high demand for maintaining physical health and improving work efficiency. If nutritional products can provide effective health care, such as enhancing immunity and antioxidant capacity (34), high-pressure working groups will be more likely to purchase them. From the perspective of the function in stress relief, high-pressure working groups usually face greater pressure from work and psychology. If nutritional products can effectively release stress, such as improving sleep quality and reducing anxiety and fatigue (35), high-pressure working groups are more motivated to purchase them. From the perspective of convenience and accessibility, high-pressure working groups are usually tight on time, and have a high demand for convenience and accessibility. If nutritional products are available in convenient ways to use and carry (36), the high-pressure working groups are more likely to purchase them. Last, from the perspective of word-of-mouth and trust, the high-pressure working groups have a high demand for word-of-mouth and trust in products. If the nutritional products have a good reputation and have been trusted by the market, the high-pressure work group will be more motivated to purchase them.

To sum up, the perceived value of nutritional products has an important impact on the purchase intention of high-pressure working groups. High-pressure working groups usually have higher needs for health care and stress relief, so if nutritional products can provide effective functions, together with convenience, good reputation and trust, they can better satisfy the needs of high-pressure working groups, thus influencing their purchase intention.

Based on the above analyses, the following hypothesis was proposed.

*H2*: Perceived value mediates the relationship between psychological ownership over nutritional products and purchase intentions of high-pressure working groups.

### 2.4 The moderating role of nutritional awareness

Nutritional awareness refers to individuals’ perception of and attention to nutrition, which includes knowledge of nutrition, awareness of their own nutritional needs, and attention to healthy diet (37, 38). Increased nutritional awareness can lead to improved dietary habits and better health among individuals who are more aware of their nutritional intake.

In modern society, more and more people are paying attention to their nutritional intake with improved living standards and health awareness (39). Increased nutritional awareness is essential for the individuals’ health. By being aware of their nutritional needs, individuals can make better food choices to maintain good health (40). In addition, increased nutritional awareness can lead to greater concern for food quality and safety, avoiding harmful substances and reducing the risk of chronic diseases (41).

Nutritional awareness plays an important role in the moderation of psychological ownership over nutritional products. When individuals’ nutritional awareness increases, they pay more attention to their own nutritional needs and pay more attention to healthy diets, which enhances their sense of identity and ownership of nutritional products (42). Individuals will be more willing to purchase and use nutritional products that meet their needs for nutrients, thus increasing their psychological ownership over nutritional products. On the other hand, nutritional awareness also moderates the purchase intentions of high-pressure working groups. High-pressure working groups usually face greater work pressure and mental stress, and are prone to malnutrition. When these people are aware of their nutritional problems, they will be more concerned about their nutritional intake and more willing to purchase and use nutritional products to improve their nutritional status (43). Therefore, increased nutritional awareness can lead to increased purchase intention and use of nutritional products among the high-pressure working groups.

To sum up, nutritional awareness reflects the knowledge of individuals and the importance they attach to nutrition. It is crucial to individuals’ health, and can prompt individuals to pay more attention to their own nutritional intake, improve their dietary habits and health. Better nutritional awareness can also moderate the psychological ownership over nutritional products and the purchase intention of high-pressure working groups, leading individuals to be more willing to buy and use nutritional products that meet their needs.

Based on the above analyses, the following hypothesis was proposed.

*H3*: Nutritional awareness moderates the relationship between psychological ownership over nutritional products and purchase intentions of high-pressure working groups.

**Figure 1:**
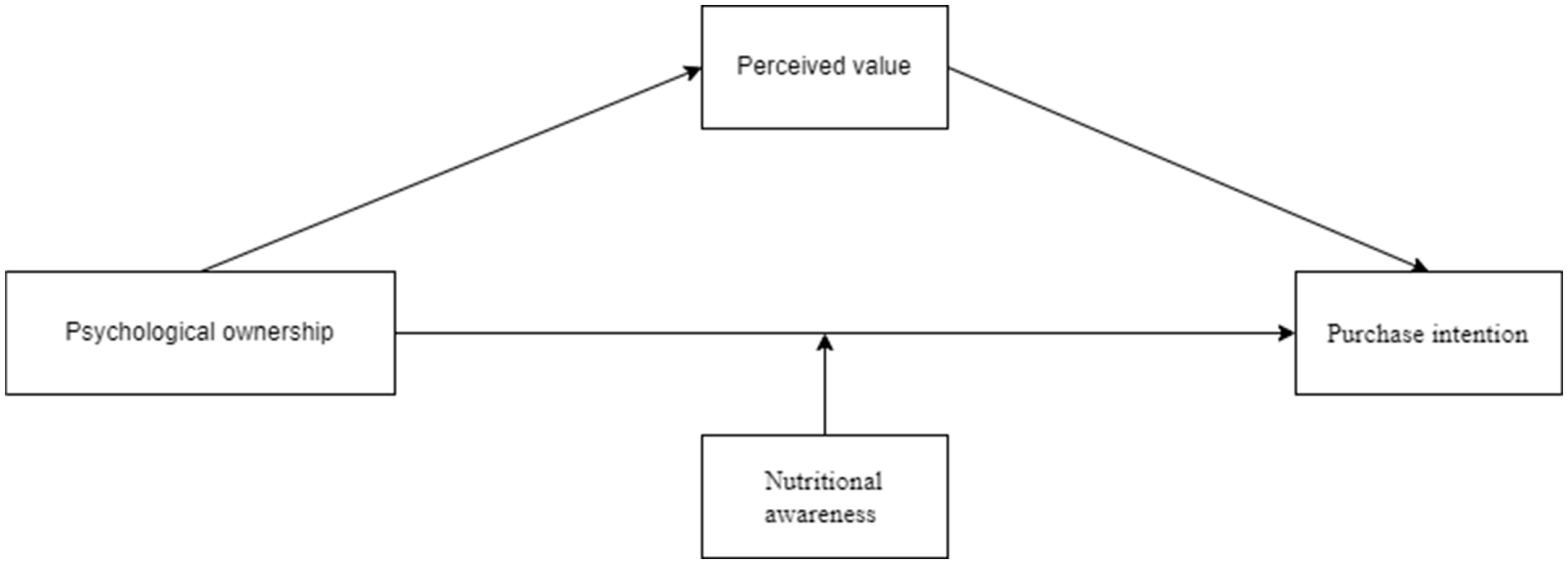
Theoretical model frame diagram.

The conceptual model is presented in Figure 1.

## 3 Overview of experiments

In order to validate the above three hypotheses, three related experiments were conducted. Experiment 1 explored the impact of psychological ownership over nutritional products on the purchase intention of high-pressure working groups, which verified H1; Experiment 2 analyzed the mediating role of perceived value in the relationship between the psychological ownership over nutritional products and the purchase intention of high-pressure working groups, which verified H2; and Experiment 3 investigated that nutritional awareness moderated the relationship between the psychological ownership over nutritional products and the purchase intention of high-pressure working groups, which verified H3. For better manipulation on the psychological ownership over nutritional products and the purchase intention of high-pressure working groups, we used different scenarios and stimulus pictures from both domestic and international sources.

The research framework associated with these three experiments is shown in Table 1.

**Table 1:** The specific research framework of three experiments.

Experiment	Experiment1	Experiment 2	Experiment 3
Purpose	To test for main effects (H1)	To test the mediating effect of perceived value (H2)	To test the moderating effect of nutritional awareness (H3)
Independent variable	Psychological ownership	Psychological ownership	Psychological ownership
Dependent variable	Purchase intention	Purchase intention	Purchase intention
G*Power predicts sample size	176	68	68
Actual sample size	285	280	265
Mediators	–	Perceived value	–
Moderator	–	–	Nutritional awareness
Measurement items	What’s your age?	What’s your age?	What’s your age?
	What is your gender?	What is your gender?	What is your gender?
	What’s your educational background?	What’s your educational background?	What’s your educational background?
	When you look at the nutritional products in the picture above, do you agree that your mood is happy?	When you see the above products, do you agree that your mood is happy?	When you see the above nutritional health products, do you agree that your mood is happy?
	Do you agree that you would like to purchase the nutritional products	Do you agree that you can feel a strong nutritional value in the above product?	Do you agree that whether a food is healthy or not has little impact on your choices?
		Do you agree that the nutritional value of the above product appeals to you?	Do you agree that you are particular about the nutritional health of the food you eat?
		Do you agree that you would like to purchase the nutritional products	Do you agree that you always follow a healthy and balanced diet?
			Do you agree that it is important for you to know how to eat healthily?
			Do you agree that you would like to purchase the nutritional products?
Methods	ANOVA	ANOVA PROCESS 4	ANOVA PROCESS 1
Results	Supported H1	Supported H2	Supported H3

## 4 Pilot study

Pilot study was done to select those nutritional products that met the criteria in people’s minds and were applicable to the three experiments. 150 participants were recruited at Credamo^1^. To enhance the accuracy and authenticity of the experimental results, all participants were informed that they were invited to join in a nutritional product competition hosted by us, to select the nutritional product that after their heart. Then, we introduced those exhibited nutritional products and asked participants, “The picture shows the nutritional product chosen for this competition, do you agree that this nutritional product is the nutritional product after your heart?” (1 = strongly disagree, 7 = strongly agree). Last, we chose three nutritional products that the participants have picked for the next experiments. All the nutraceuticals for which the three experiments were performed are shown in Table 2.

**Table 2:** Results of the pilot study.

Nourishment Image	Product description	*M*	SD	Selection for experiments
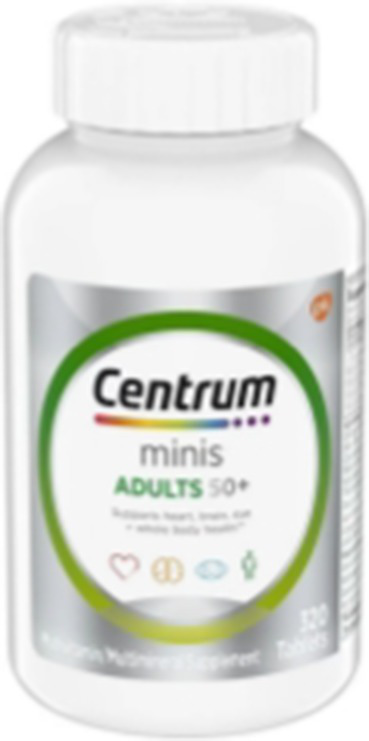	Centrum — Nutritional element tablets for the elderly	6.55	1.309	Yes. (Experiment 1)
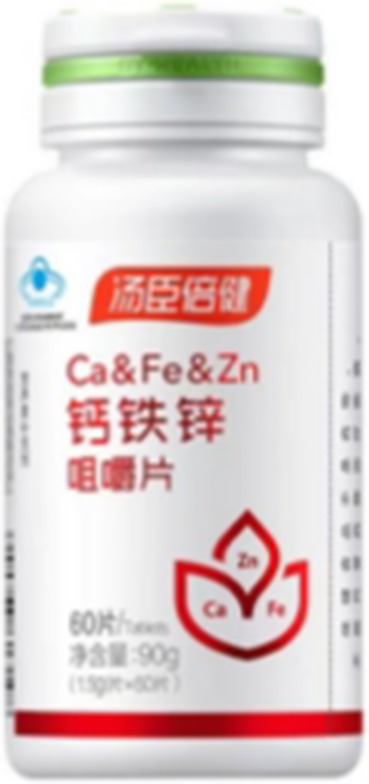	Tomson Beijian — calcium, iron and zinc chewable tablets	6.49	1.374	Yes. (Experiment 2)
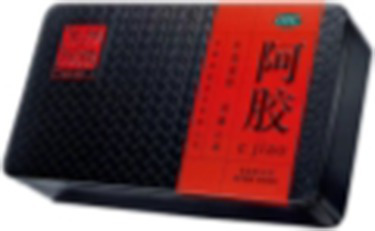	Donkey-hide gelatin	6.51	1.299	Yes. (Experiment 3)
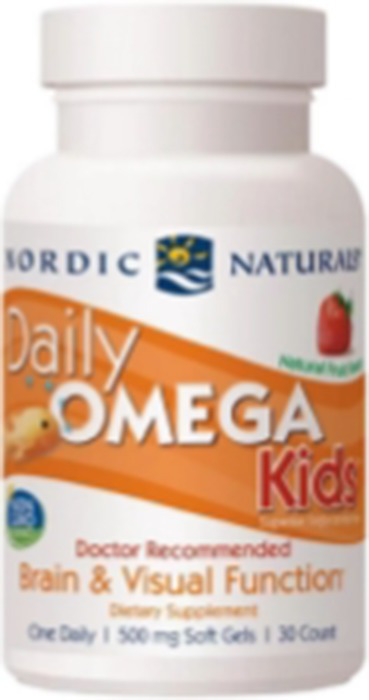	Nordic naturals fish oil	5.97	1.815	No
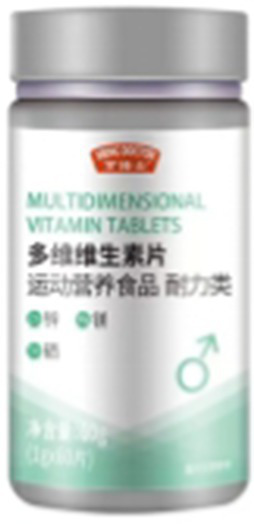	Dr. Heng — multivitamin tablets	5.30	2.097	No
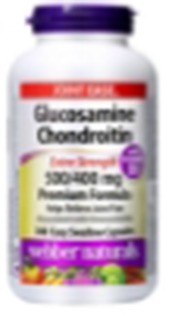	Weibaijian — Healthy bone supreme glucosamine chondroitin capsule	5.25	2.057	No
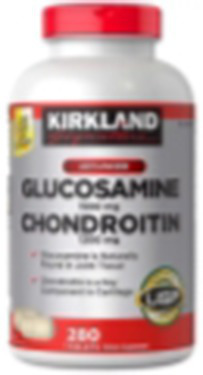	Kirkland glucosamine and chondroitin	5.19	2.081	No

## 5 Experiment 1: main effect of psychological ownership over nutritional products on purchase intentions of high-pressure working groups

### 5.1 Experimental design and data analysis

The objective of Experiment 1 was to investigate the main effect of psychological ownership of nutritional products on the purchase intention of individuals living under high stress, while also testing Hypothesis H1. Variance analysis was performed using the SPSS 26.0 software, with psychological ownership as the independent variable and purchase intention as the dependent variable. We conducted a one-way between subjects ANOVA (psychological ownership over nutritional products: with vs. without). We randomly recruited 285 participants at Credamo, of which 148 (51.9%) were male and 137 (48.1%) were female. The age distribution of the participants was 75 (26.3%) aged 18–25 years, 80 (28.1%) aged 26–40 years, 69 (24.2%) aged 41–60 years, and 61 (21.4%) aged 61 years and above. See Table 3 for details. All participants were then assigned to the same scenario of large pharmacy. One hundred forty-two participants in the low psychological ownership group had the general nutritional products as stimulus material and 143 participants in the high psychological ownership group had customized nutritional products as stimulus material.

**Table 3:** Summary of demographic information.

Characteristics of subjects		Experiment 1	Percentage	Experiment 2	Percentage	Experiment 3	Percentage
Gender	Male	148	51.9%	136	48.6%	131	49.4%
	Female	137	48.1%	144	51.4%	134	50.6%
	18–25 years old	75	26.3%	74	26.4%	68	25.7%
	26–40 years old	80	28.1%	87	31.1%	76	28.7%
	41–60 years old	69	24.2%	68	23.9%	60	22.6%
	Over 60 years old	61	21.4%	52	18.6%	61	23.0%
Education background	Primary school	5	1.8%	10	3.6%	9	3.4%
	Middle school	23	8.1%	19	6.8%	19	7.2%
	High school and technical secondary school	23	8.1%	15	5.4%	18	6.8%
	Junior college	42	14.7%	61	21.8%	55	20.8%
	Undergraduate college	67	23.5%	62	22.1%	60	22.6%
	Master degree candidate	66	23.2%	72	25.7%	69	26.0%
	Doctor-postgraduate	59	20.7%	41	14.6%	35	13.2%

**Figure 2:**
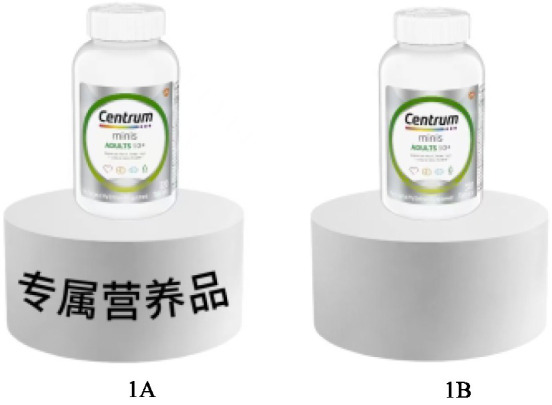
Stimulus materials used in experiment 1.

All participants were led to imagine that they were shopping in a large pharmacy with a special sales area. Participants in the low psychological ownership group were presented with the general nutritional products being sold in this area, while those in the high psychological ownership group were presented with the customized nutritional products being sold in this area, and learnt that the nutritional products could be tailored to suit different situation. We assigned a value of 2 to the low psychological ownership group and a value of 1 to the high psychological ownership group, to differentiate their psychological ownership over the nutritional products. Participants were then asked, “Do you agree that you would like to purchase the nutritional products” (1 = strongly disagree, 7 = strongly agree) to measure their purchase intention. The stimulus material for Experiment 1 was shown as in Figure 2.

Referring to the study of Tan et al. (44), the emotions of high-pressure working groups affect their purchase decision-making, by influencing their shopping experience. Therefore, we performed the manipulation on the emotions of the high-pressure working groups. Participants were asked, “Do you agree that your emotions were pleasant when you saw the nutritional products customized for you?” (1 = strongly disagree, 7 = strongly agree) to measure their emotional state. After that, we collected the basic demographic information of the participants.

### 5.2 Experimental results

There was a main effect test. We conducted a one-way ANOVA with the psychological ownership over the nutritional products as the independent variable and purchase intention of high-pressure working groups as the dependent variable. The experimental results showed that participants’ purchase intention in the high psychological ownership group (*M* = 5.36, SD = 1.041) was significantly higher than that in the low psychological ownership group (*M* = 5.02, SD = 1.297), *F*(1, 283) = 5.888, *p* = 0.016. H1 was validated.

There was a control variable analysis. Referring to the study of Tan et al. (44), the emotions of the high-pressure working groups affect their consumption behavior. We analyzed the impact of psychological ownership over nutritional products on the purchase intention of the high-pressure working groups again, by taking the emotions of the high-pressure working groups as a covariate. Based on the ANCOVA, there is no significant impact of emotions on the purchase intention of the high-pressure working groups [*F*(1, 283) = 7.632, *p* = 0.006]. Therefore, the alternative explanation of emotions of the high-pressure working groups has been excluded. Again, H1 was validated.

### 5.3 Discussion

Experiment 1 validated the main effect of psychological ownership over nutritional products on the purchase intention of high-pressure working groups. The results showed that psychological ownership over nutritional products have a direct positive impact on the purchase intention of the high-pressure working groups. At the same time, we also affirmed that the emotions of the high-pressure working groups have no significant impact on their purchase intention. However, despite the above experimental findings, Experiment 1 failed to further analyze the internal mechanisms and boundary conditions in the relationship between psychological ownership over nutritional products and purchase intention of high-pressure working groups. In order to fill the gaps, Experiment 2 introduced the perceived value as a mediating variable, so as to better explore the impact of psychological ownership over nutritional products on purchase intention of high-pressure working groups.

## 6 H2: the mediating role of perceived value

### 6.1 Experimental design and data analysis

The purpose of Experiment 2 was to verify the mediating role of perceived value between the psychological ownership of nutritional products and the purchase intention of high-pressure working groups, while also testing Hypothesis H2. Variance analysis was conducted using the SPSS 26.0 software for calculations, with ‘psychological ownership’ as the independent variable and ‘purchase intention’ as the dependent variable. The mediating model employed in the analysis was Process Model 4, utilizing ‘perceived value’ as the mediating variable. We randomly recruited 280 participants at Credamo, of which 136 (48.6%) were males and 144 (51.4%) were females. The age distribution of the participants was 74 (26.4%) aged 18–25 years, 87 (31.1%) aged 26–40 years, 67 (23.9%) aged 41–60 years, and 52 (18.6%) aged 61 years and above. See Table 3 for details. All participants were randomly assigned to the same scenario of large nutritional product fair. The 140 participants in the low psychological ownership group were presented with general nutritional products; the 140 participants in the high psychological ownership group were presented customized nutritional products.

**Figure 3:**
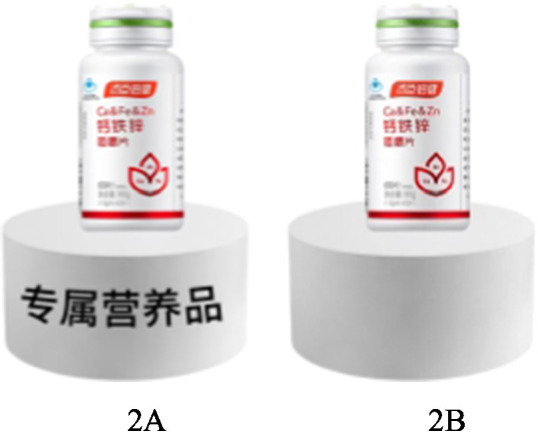
Stimulus materials used in experiment 2.

All participants were led to imagine that they were involved in a large nutritional product fair. Participants in the low psychological ownership group were told that there were various nutritional products in the fair, when they saw a nutritional product in the venue. Participants in the high psychological ownership group were told that one of the nutritional products had been successfully developed and manufactured by their team. As the main members of the team, participants in the high psychological ownership group were invited to serve as a product commentator to help select products for the fair. After the fair, the organizers will gift them a health product. We distinguished the psychological ownership over nutritional products, by assigning a value of 2 to the low psychological ownership group and a value of 1 to the high psychological ownership group. Participants were then asked, “Do you agree that you can feel a strong nutritional value in the above product?” and “Do you agree that the nutritional value of the above product appeals to you?” (1 = strongly disagree, 7 = strongly agree) to measure their perceived value, with questions adapted from a scale of Han and Yoon (45). Last, participants were asked, “Do you agree that you would like to purchase the nutritional products” (1 = strongly disagree, 7 = strongly agree) to measure their purchase intention. The stimulus material for Experiment 2 was shown as in Figure 3.

### 6.2 Experimental results

There was a main effect test. We conducted a one-way ANOVA with psychological ownership over nutritional products as the independent variable and purchase intention of the high-pressure working groups as the dependent variable. The experimental results showed that the purchase intention of the high psychological ownership group (*M* = 6, SD = 1.545) was significantly higher than that of the low psychological ownership group (*M* = 4.15, SD = 1.388), *F*(1, 278) = 111.031, *p* < 0.001. Verifying H1.

**Figure 4:**
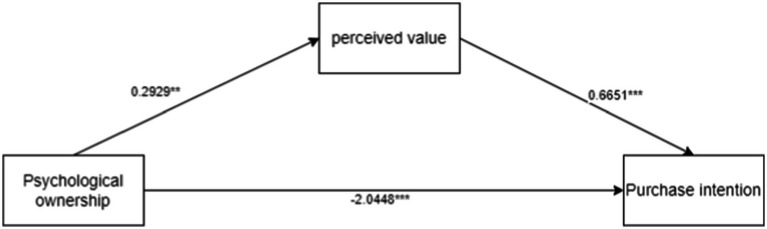
Experiment 2: mediating effect path map.

There was a mediating effect analysis. We took psychological ownership over nutritional products as the independent variable, perceived value as the mediator, and purchase intention of high-pressure working groups as the dependent variable, employing process model 4 to explore the perceived value, which mediated the relationship between psychological ownership over nutritional products and purchase intention of high-pressure working groups [Bootstrap sample: 5000; (46)]. The results showed that psychological ownership over nutritional products had a significantly positive impact on perceived value (*β* = 0.2929, *p* = 0.005, 95% CI = [0.089 to 0.4967]); psychological ownership over nutritional products had a significantly negative impact on purchase intentions of the high-pressure working groups (*β* = −2.0448, *p* < 0.0001, 95% CI = [−2.3678 to −1.7217]); perceived value had a significantly positive impact on purchase intention of the high-pressure working groups (*β* = 0.6651, *p* < 0.0001, 95%CI = [0.4806 ~ 0.8495]). The mediation process of psychological ownership over nutritional products-perceived value-purchase intention of high-pressure working groups was significant (*β* = 0.1948, SE = 0.0867, 95% CI = [0.0507 ~ 0.3834]). Therefore, perceived value fully mediated the relationship between psychological ownership over nutritional products and purchase intention of high-pressure working groups. H2 was validated. The mediation model results of Experiment 2 are shown in Figure 4.

### 6.3 Discussion

Experiment 2 confirmed the mediating role of perceived value in the relationship between psychological ownership over nutritional products and purchase intention of the high-pressure working groups. The higher the perceived value of high-pressure working groups, the more likely they were to have psychological ownership over the nutritional product, which enhanced their purchase intention. Despite the above findings, Experiment 2 only discussed the mediating role between psychological ownership over nutritional products and purchase intention of the high-pressure working groups, but failed to further discuss whether there was a moderating role between the two. Therefore, Experiment 3 introduced nutritional awareness and attempted to explore its moderating role in the relationship between psychological ownership and purchase intention of the high-pressure working groups.

## 7 H3: the moderating role of nutritional awareness

### 7.1 Experimental design and data analysis

The purpose of Experiment 3 was to investigate the moderating effect of nutritional awareness on the relationship between psychological ownership of nutritional products and purchase intention among high-pressure working groups, while also testing Hypothesis H3. We conducted a 2 (psychological ownership over nutritional products: with vs. without) × 2 nutritional awareness (high vs. low) ANOVA. Variance analysis was executed using the SPSS 26.0 software, with ‘psychological ownership’ as the independent variable and ‘purchase intention’ as the dependent variable. In this analysis, the moderation model utilized Process Model 1, with ‘nutritional awareness’ serving as the moderating variable. Two hundred sixty-five participants were randomly recruited at Credamo, of which 131 (49.4%) were male and 134 (50.6%) were female. The age distribution of the participants was 68 (25.7%) aged 18–25 years, 76 (28.7%) aged 26–40 years, 60 (22.6%) aged 41–60 years and 61 (23%) aged 61 years and above. See Table 3 for details. All participants were randomly assigned to the same scenario of competitive reality show, where 128 participants in the low psychological ownership group were presented with general nutritional products; 137 participants in the high psychological ownership group were presented with customized nutritional products.

**Figure 5:**
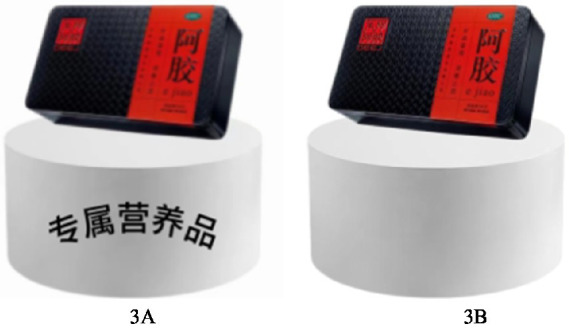
Stimulus materials used in experiment 3.

All participants were led to imagine that they were participating in a competitive reality show. Participants in the low psychological ownership group were told that the ultimate reward was a nutritional product. However, they failed the game. The participants in the high psychological ownership group were told that the ultimate reward was a nutritional product that they were going to buy. And they successfully survived the game, making it their own nutritional product. We assigned a value of 2 to the low psychological ownership group and a value of 1 to the high psychological ownership group, to differentiate their psychological ownership over the nutritional products. Participants were then asked, “Do you agree that whether a food is healthy or not has little impact on your choices?,” “Do you agree that you are particular about the nutritional health of the food you eat?,” “Do you agree that you always follow a healthy and balanced diet?” and “Do you agree that it is important for you to know how to eat healthily?” (1 = Strongly Disagree, 7 = Strongly Agree). These four questions were adapted from a scale of van Dillen et al. (47), to measure participants’ nutritional awareness. Last, participants were asked, “Do you agree that you would like to purchase the nutritional products” (1 = strongly disagree, 7 = strongly agree) to measure their purchase intention. After that, we collected the basic demographic information of the participants. The stimulus material for Experiment 3 was shown as in Figure 5.

### 7.2 Experimental results

There was a main effect test. We conducted one-way ANOVA with psychological ownership over nutritional products as the independent variable and purchase intention of the high-pressure working groups as the dependent variable. The experimental results showed that the purchase intention of the participants in the high psychological ownership group (*M* = 6.3, SD = 0.977) was significantly higher than that of the low psychological ownership group (*M* = 5.69, SD = 1.608), *F*(1, 263) =14.089, *p* < 0.001. H1 was validated.

There was a manipulation check. We divided the participants into two groups, according to the mean value of nutritional awareness, with high nutritional awareness being assigned a value of 2 and low nutritional awareness being assigned a value of 1. Then we conducted a one-way ANOVA with “nutritional awareness” as the independent variable and the purchase intention of high-pressure working groups as the dependent variable. The results showed that the purchase intention of the participants with high nutritional awareness (*M* = 5.24, SD = 1.745) was significantly higher than that with low nutritional awareness (*M* = 6.52, SD = 0.628), and *F*(1, 263) = 70.597, *p* < 0.001. The manipulation in experiment 4 was successful.

**Figure 6:**
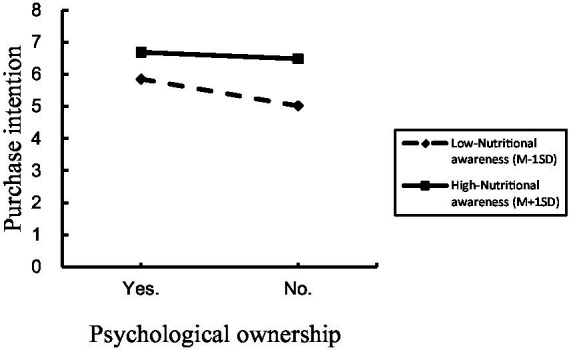
H3: the moderating role of nutritional awareness.

There was a moderating effect analysis. We took psychological ownership over nutritional products as the independent variable, nutritional awareness as the moderating variable, and purchase intention of high-pressure working groups as the dependent variable. Process model 1 was employed to analyze the moderating role of nutritional awareness, as well as the interaction effect of nutritional awareness and psychological ownership over nutritional products on the purchase intention of the high-pressure working groups [Bootstrap sample: 5000; (46)]. The results showed that psychological ownership over nutritional products had a significantly negative impact on the purchase intention of the high-pressure working group (*β* = −0.5201, *p* < 0.001, 95% CI = [−0.8116 ~ −0.2287]), and nutritional awareness had a significantly positive impact on the purchase intention of the high-pressure working groups (*β* = 0.5491, *p* < 0.001, 95% CI = [0.411 ~ 0.6871]), and the interaction effect between psychological ownership over nutritional products and nutritional awareness had a significantly positive impact on the purchase intention of the high-pressure working groups (*β* = 0.2991, *p* = 0.034, 95% CI = [0.0224 ~ 0.5758]). It indicated that nutritional awareness moderates the relationship between psychological ownership over nutritional products and purchase intention of high-pressure working groups. H3 was validated. The mediation model results of Experiment 3 are shown in Figure 6.

### 7.3 Discussion

Experiment 3 verified that the interaction effect between nutritional awareness and psychological ownership has a significant impact on the purchase intention of the high-pressure working groups. The experimental results show that regardless of the psychological ownership over nutritional products, high-pressure working groups with high nutritional awareness have significantly higher purchase intention than that with low nutritional awareness. The nutritional products that are compatible with consumers’ nutritional value and health consciousness could motivate their psychological ownership over nutritional products, so as to make them to purchase nutritional products that meet nutritional needs and health concepts.

## 8 General discussion

By exploring the function of psychological ownership over nutritional products and its impact on the purchase intention of high-pressure working groups, this study fills the gap in the existing literature. The findings of this study are consistent with the research conducted by Hoeijmakers et al. (48) which demonstrated that early-life stress can impact an individual’s nutritional status, reflecting the issue that high stress levels can lead to malnutrition.

At the same time, this study also explores the mediating role of perceived value and the moderating role of nutritional awareness, in the relationship between the psychological ownership over nutritional products and the purchase intention of high-pressure working groups, enriching the knowledge on the perceived value theory.

### 8.1 Theoretical contributions

#### 8.1.1 Discussion based on Hypothesis H1 validation

This study found that psychological ownership over nutritional products had a significant impact on the purchase intention of the high-pressure working groups. The high-pressure working groups tend to regard the nutritional product as their psychological property, which enhances the purchase intention. This finding provides important theoretical support for the nutritional products marketing. Merchants can increase the purchase intention of the high-pressure working groups by motivating their psychological ownership over the nutritional products.

This study found that psychological ownership over nutritional products is effective in promoting the purchase intention of high-pressure working groups. In previous studies, psychological ownership and work-related stress have been identified as factors that can influence consumer behavior (49, 50). However, few studies have linked these two factors together and explored how psychological ownership over nutritional products affects the purchase intention of high-pressure working groups. Although nutritional products have been proven to be one of the important factors influencing people’s health status (51), the impact of psychological ownership over nutritional products on the purchase intention of high-pressure working group has not yet been thoroughly investigated. Therefore, this study responded to the appeal of recent research, by emphasizing the important impact of psychological ownership over nutritional products (24). By integrating these factors into the impact of psychological ownership over nutritional products on the purchase intention of high-pressure working groups, this study advanced knowledge in the field of nutritional product consumption among high-pressure working groups. On the one hand, this study extended the literature on psychological ownership over nutritional products and consumption behavior of high-pressure working groups (52); on the other hand, by highlighting new antecedents of high-pressure working groups (20), the findings provide priori knowledge and a better understanding of how psychological ownership over nutritional products affect purchase intentions of high-pressure working groups.

#### 8.1.2 Discussion based on Hypothesis H2 validation

This study verified the mediating role of perceived value in the relationship between psychological ownership over nutritional products and purchase intention of high-pressure working groups. The expectations and satisfaction of the high-pressure working group can enhance the perceived ownership and control of high-pressure working groups for certain brand, which further enhances their purchase intention. This finding enriches the knowledge on perceived value theory and provides important theoretical support for the nutritional products marketing.

Based on psychological ownership theory, this study aimed to investigate the mediating role of perceived value in the relationship between psychological ownership over nutritional products and purchase intention of high-pressure working groups. Previous research has demonstrated the effectiveness of nutritional product advertisements in influencing consumer behavior, particularly in terms of purchase intention and psychological ownership over nutritional products (52). However, the mediating role of the perceived value of high-pressure working group on their purchase intention has not been fully validated. Therefore, this study advanced research in this area, by exploring the perceived value of high-pressure working groups and its impact on psychological ownership over nutritional products and purchase intention. Although previous research has supported the ability of nutritional product advertisements to evoke consumer behavior (53), the mediating role of perceived value on the high-pressure working groups, and its impact on their purchase intentions, remains unknown. Therefore, in other words, it is also unknown whether the perceived value of nutritional products could mediate the relationship between psychological ownership over nutritional products and purchase intention of the high-pressure working groups. This study was one of the early attempts to validate the mediating role of perceived value in the relationship between psychological ownership over nutritional products and the purchase intention of high-pressure working groups, based on psychological ownership theory. This study found that the perceived value of nutritional products among high-pressure working groups affects their psychological ownership of nutritional products, which in turn affects their purchase intentions. This finding has important theoretical and managerial implications for developing and promoting the marketing strategies of nutritional products.

#### 8.1.3 Discussion based on Hypothesis H3 validation

This study also verified the moderating effect of nutritional awareness. The high-pressure working groups pay more attention to the nutritional value and health factors of products, which could further increase their purchase intention. Merchants can attract the high-pressure working groups, by emphasizing the nutritional value and health factors of products.

This study identified an important moderating role for nutritional awareness. Previous research has shown that nutritional awareness can influence individuals’ perceptions and attitudes toward nutritional products, which in turn affects their psychological ownership over nutritional products (37). On the one hand, individuals with high nutritional awareness pay more attention to their nutritional needs and are more likely to perceive nutritional products as beneficial to their health, and therefore are more likely to develop the psychological ownership over nutritional products. On the other hand, high-pressure work groups are often prone to malnutrition due to factors such as high work pressure and tight schedules (54). These individuals have a more urgent need for nutritional products and therefore their purchase intention is relatively high. Nutritional awareness may have a moderating effect on the purchase intention of high-pressure working groups. Individuals with high nutritional awareness pay more attention to their nutritional needs, so they are more likely to be aware of their nutritional deficiencies and are more willing to purchase nutritional products to meet their own needs. On the contrary, individuals with low nutritional awareness are less likely to be aware of their nutritional deficiencies, and therefore their purchase intention for nutritional products may be relatively low. It can be seen that nutritional awareness plays an important moderating role in the relationship between psychological ownership over nutritional products and purchase intention of high-pressure working groups (39). This theoretical contribution extended the perspective of nutritional product consumption behavior, by providing new explanations and theoretical support for the purchase intention of high-pressure working groups. Further research could explore how to increase nutritional awareness among high-pressure work groups, so as to promote their purchase intention and consumption behavior of nutritional products.

In summary, the contribution of this study focuses on the exploration of the psychological ownership over nutritional products, and how it combines with the perceived value and nutritional awareness, to affect the purchase intention of the high-pressure working groups. This study not only devotes to an in-depth understanding of nutritional product marketing and the consumer behavior of the high-pressure working groups, but also provides valuable measures for merchants to increase the purchase intentions of the high-pressure working group.

### 8.2 Managerial implications

#### 8.2.1 Managerial implications derived from Hypothesis H1

The findings of this study provided valuable insights into enhancing the purchase intentions of high-pressure working groups for nutritional products. Most of the high-pressure working groups lack the perception of psychological ownership over nutritional products, which may affect their purchase intention. Therefore, retailers should consider facilitating the purchase intention of high-pressure working groups, by increasing their perceptions of psychological ownership over nutritional products (55). Specifically, retailers can help the high-pressure working groups understand the importance of nutritional products to their health and work performance, by providing relevant education and information. In addition, retailers can collaborate with professional dietitians to provide personalized nutritional counseling and advice to the high-pressure working groups to increase their purchase intention, by helping them understand their needs for nutritional products (56). With these managerial implications, retailers can better meet the nutritional needs of the high-pressure working groups, so as to improve their productivity and quality of life.

#### 8.2.2 Managerial implications derived from Hypothesis H2

This study found that perceived value mediated the relationship between psychological ownership over nutritional products and purchase intentions of high-pressure working groups. According to the results, retailers can increase the purchase intentions of high-pressure working groups by enhancing their perceived value of nutritional products (57). Specifically, retailers can conduct marketing activities to convey the importance and benefits of nutritional products to the high-pressure working groups, in order to increase their perceptions and acceptance. In addition, retailers can provide customized nutritional product and personalized purchasing options to meet the specific needs and preferences of the high-pressure working groups (58). Through these measures, retailers can enhance the psychological ownership over nutritional products among the high-pressure working groups, thereby promoting their purchase intention.

#### 8.2.3 Managerial implications derived from Hypothesis H3

It was found that the purchase intention of high-pressure working groups is moderated by the nutritional value, when they are facing work stress and health problems. Thus, nutritional products with high nutritional value are more likely to attract the purchase intention of high-pressure working groups, by satisfying their needs. Therefore, retailers and brands should emphasize the high nutritional value of nutritional products to motivate this group of people (59). One of the most important things is to understand which high-pressure working groups are of greater nutritional awareness. The needs and awareness of high-stress working groups may vary across industries and occupations. Therefore, retailers and brands need to know more about the characteristics and needs of different groups in order to pointedly perform their marketing activities and product promotions. Through market research and data analysis, retailers can understand the degree of nutritional awareness and purchasing preferences of different groups with high working pressure. For example, some high-pressure working groups may focus more on nutritional products to improve work efficiency and cope with stress, while others may be more concerned with physical health and maintenance. Understanding these differences can help retailers pinpoint their target groups, develop nutritional products that meet their needs, and formulate marketing strategies accordingly. In summary, understanding which high-pressure groups are more nutritionally aware is of utmost importance. Retailers and brands can carry out targeted market research and data analysis, to understand the characteristics and needs of different high-pressure working groups. It can help to accurately locate the target groups, develop nutritional products that meet their needs and formulate corresponding marketing strategies, thus promoting the purchase intention of high-pressure working groups for nutritional products, thus achieving the development and growth of the nutritional products market.

### 8.3 Limitations and future research

This study acknowledges the following limitations. First, this study has a limitation of external validity (60), as it only examined the relationship between psychological ownership of nutritional products and the purchase intentions of high-pressure working groups. Future research could consider expanding the sample to include more high-pressure working groups with different occupational backgrounds and work environments to enhance the external validity. Second, this study only focused on the impact of psychological ownership over nutritional products on purchase intention, future research could further explore the impact of other factors among high-pressure work groups, such as personal health awareness (61), and level of work stress (62). Last, this study is only a preliminary exploration of the relationship between psychological ownership over nutritional products and the purchase intentions of high-pressure working groups, and future research could delve deeper into the mechanisms and influencing factors, to provide more specific suggestions for practice.

## Data availability statement

The raw data supporting the conclusions of this article will be made available by the authors, without undue reservation.

## Author contributions

BL: Data curation, Investigation, Project administration, Supervision, Writing – original draft, Writing – review & editing. DY: Investigation, Methodology, Software, Visualization, Writing – original draft, Writing – review & editing. FT: Conceptualization, Formal analysis, Investigation, Writing – original draft, Writing – review & editing. DS: Data curation, Investigation, Supervision, Writing – original draft, Writing – review & editing. JL: Data curation, Investigation, Validation, Writing – original draft, Writing – review & editing.

## Footnotes

^1^https://www.credamo.com
